# INTEREST GROUP SESSION—TECHNOLOGY AND AGING: THE POTENTIAL AND PITFALLS OF EXTENDED REALITY SOLUTIONS FOR SUPPORTING AND ASSESSING OLDER ADULTS

**DOI:** 10.1093/geroni/igz038.892

**Published:** 2019-11-08

**Authors:** Neil Charness, Balaji Narasimhan

**Affiliations:** 1 Florida State University, Tallahassee, Florida, United States; 2 Iowa State University, Ames, Iowa, United States

## Abstract

Extended Reality (XR), which encompasses Virtual Reality (VR) and Augmented Reality (AR), holds a great deal of promise for improving the health and well-being of older adults. These opportunities include providing rehabilitation, physical exercise, skills training, leisure opportunities, and support for instrumental activities of daily living. Further, XR presents novel assessment opportunities. This session will explore the potential of XR solutions, and also crucial barriers to XR implementation, adoption, and engagement, particularly with respect to the “digital divide.” Some older adults, for a number of reasons, experience greater challenges adopting and using newer technologies. This session will start with a broad overview of issues related to XR solutions and will identify critical research needs, with an emphasis on the needs of older adults. This will be followed by a presentation of older adults’ perceptions of XR using data derived from a large, nationally representative sample. While some older adults reported not being ready for XR solutions, many older adults reported being willing to accept them to support optimal aging. Next, a study is presented that directly compares older adults’ perceptions of presence and immersion in virtual spaces. Using VR to assess wayfinding and navigation abilities of older adults is discussed next. The final talk will present VR usability issues derived from interview and focus group data. The session discussant will bring an interdisciplinary perspective to these important issues.

